# Delayed Guillain-Barré Syndrome after Bariatric Surgery: A Report of Three Cases

**DOI:** 10.1155/2018/8413206

**Published:** 2018-12-20

**Authors:** Alana H. Sunbol, Sarah Almaghrabi, Shahd J. Al Aslany, Mashael M. Aldairi, Sara A. Makhdum, Nora Trabulsi, Mohammed A. Almekhlafi, Mohammed Nassif

**Affiliations:** ^1^Faculty of Medicine, King Abdulaziz University, P.O. Box 80200, Jeddah 21589, Saudi Arabia; ^2^Department of Surgery, Faculty of Medicine, King Abdulaziz University, P.O. Box 80200, Jeddah 21589, Saudi Arabia; ^3^Department of Neurology, Faculty of Medicine, King Abdulaziz University, P.O. Box 80200, Jeddah 21589, Saudi Arabia

## Abstract

Surgeries carry a risk of complications. Polyneuropathies, including Guillain-Barré syndrome (GBS), are potential complications of bariatric surgery. The incidence of these conditions is expected to increase as these surgeries become increasingly popular. We present a case report of three patients who developed a polyneuropathy after bariatric surgery. GBS was diagnosed in each patient, with nutritional deficiencies being suspected as a contributing factor. All patients began a 5-day intravenous immunoglobulin course in addition to receiving rehabilitative support, multivitamins, intravenous thiamine, vitamin D (therapeutic dose), and selenium. The patients' symptoms improved but did not completely resolve. GBS can be a complication of bariatric surgery. Although a clear cause-effect relationship cannot be established for the present cases, the cumulative literature on the subject suggests that it is important to include it as a potential risk when counseling patients for such surgeries.

## 1. Introduction

The prevalence of obesity has been on the rise worldwide over the last few decades. Obesity is associated with clinical and biochemical changes in various body systems [1]. In Saudi Arabia, one in three adults are obese and at least one in 10 adults are morbidly obese [2].

One evidence-based recommendation for those with morbid obesity is to undergo bariatric surgery. It is estimated that 15,000 bariatric operations are performed annually in Saudi Arabia [2]. Generally, this surgery has been shown to be one of the most effective long-term weight-reduction techniques, as it reduces cardiovascular risk and increases life expectancy. From their nutrient pass patterns, bariatric surgeries can be divided into two classes: restrictive procedures (e.g., gastric banding and sleeve gastrectomy) and gastrointestinal bypass procedures (e.g., Roux-en-Y gastric bypass and biliopancreatic diversion) [3]. Laparoscopic sleeve gastrectomy was the most performed bariatric procedure in the world in 2016 [4].

Surgeries in general carry a risk for a variety of complications. Macro- and micronutritional deficiencies may occur in some patients after bariatric surgery and can result in serious neurological sequelae, [5] including polyneuropathy. Contributing factors comprise drugs, metabolic disorders, environmental exposure, vitamin deficiencies, conversion disorders, and inflammation. Postbariatric surgery polyneuropathies are most commonly associated with deficiencies in thiamine (vitamin B_1_) and cyanocobalamin (vitamin B_12_), as well as inflammatory causes such as Guillain-Barré syndrome (GBS), myasthenia gravis, human immunodeficiency virus-associated polyneuropathy, lupus erythematosus-associated polyneuropathy, and chronic active hepatitis-associated polyneuropathy [6].

## 2. Case 1

A 20-year-old female with a body mass index (BMI) of 42 kg/m^2^, who met the National Institutes of Health criteria for bariatric surgery, underwent sleeve gastrectomy surgery in July 2016. She was discharged home without any complications. The initial 3-month postoperative period was uneventful without any nausea or vomiting. Net postoperative weight loss was 30 kg in 4 months. In early November 2016, she reported feeling flu-like symptoms (cough, sore throat, and runny nose) with a low-grade fever. Her upper respiratory symptoms improved, but 2 weeks later, symptoms rapidly progressed to bilateral ascending lower limb (LL) numbness reaching her waist. The numbness became progressively worse and she started to develop bilateral lower extremity weakness with recurrent falling attacks and severe leg pain. In addition, she developed choking attacks, hoarseness with on and off dysphagia, dyspnea, and constipation without urinary retention.

The neurologist's evaluation showed reduced distal and proximal muscle power, diminished LL reflexes, and decreased pinprick sensation up to L1 bilaterally. In addition, big toe proprioception was impaired and the patient had a decreased sense of vibration up to the knee bilaterally. There was no involvement of the upper limbs (ULs), and the cerebellum and cranial nerves were intact.

Initial laboratory test results were unremarkable, including a thyroid function test and vitamin B_12_ levels (1186 pmol/L). A lumber puncture was performed and cerebrospinal fluid (CSF) revealed the following: white blood cell count (WBC) 1 cell/cubic mm, red blood cell count (RBC) 11 cell/cubic mm, protein level 0.31 g/L, glucose level 3.4 mol/L. CSF bacterial and fungal culture results were negative. Serologic tests showed normal complement and high immunoglobulin G (16.7 g\L) levels, as well as negative antinuclear antibody (ANA) results. Because of her progression in symptoms, the patient underwent further neurological workup. Magnetic resonance imaging (MRI) scan results for the spinal cord were normal except for a high-signal density on the L1 vertebral body. The results of nerve conduction study (NCS) was normal apart from the absence of F waves, and electromyography (EMG) testing showed axonal involvement. These findings were consistent with the axonal form of GBS.

The patient required intubation and was admitted to the intensive care unit (ICU), where she received intravenous immunoglobulin (IVIG) for 5 days. Nutritional supplements, including IV thiamine, vitamin B_6_, and vitamin B_12_, were given in addition to selenium. She continued rehabilitation and physical therapy. Her symptoms gradually improved, but she required the assistance of walkers before her symptoms completely resolved.

## 3. Case 2

A 36-year-old female with diabetes mellitus, hypertension, and hypothyroidism underwent a cesarean section a few weeks prior to admission and a gastric sleeve procedure 1 year before that.

Two days before admission to hospital, the patient developed ascending numbness followed by weakness, which progressed over 2 days. It interfered with her movement, making her bed bound. She reported a 1-day history of subjective fever and diarrhea a week before the onset of symptoms, which subsided spontaneously. Weakness was not associated with double vision, shortness of breath, slurred speech, abnormal movement, or confusion. She did not have fever, diarrhea, or abdominal pain and did not have similar attacks previously.

She lost approximately 40 kg in 2 months; she did not receive any vitamin supplementation after surgery. The patient reported vomiting three to four times daily postoperatively until a month before her presentation.

Examination showed that the cranial nerves were intact with no motor or sensory abnormalities. Her UL and LL tone was normal. There was a symmetrical decrease in power in her ULs both proximally and distally. LL power was also decreased. Her reflexes were diminished but elicitable, but her plantar response was mute with various maneuvers. The sensory examination was unremarkable and her coordination was intact.

The patient was admitted to hospital as a case of postbariatric surgery myeloneuropathy or GBS. IVIG was given for a complete course of five continuous days. Thiamine loading and maintenance were given parentally. Vitamin B_12_ and D levels were within normal limits. She was discharged with persistent weakness to undergo rehabilitation. No MRI or NCS was done at admission.

She presented again with the same concern and was admitted to neurology inpatient services. An MRI scan of the entire spine was performed, which showed early dorsal spine spondylitic changes with small anterior osteophytes at the lower dorsal spine. An EMG/NCS was also done, showing pure motor neuropathy and myopathic changes at proximal muscle and a normal sensory picture, suggestive of acute motor axonal neuropathy for early Guillain-Barré syndrome.

She continued to receive vitamin supplementation and physiotherapy. Plasma exchange was discussed but declined by the family. She will require ongoing extensive neurorehabilitation at a dedicated center at King Faisal Specialist Hospital and Research Centre or King Fahad Medical City in Riyadh.

## 4. Case 3

A 22-year-old Saudi female with a case of morbid obesity (initial BMI of 43 kg/m^2^ and final postoperative BMI of 34.6 kg/m^2^), who met the National Institute of Health criteria for bariatric surgery, underwent an elective laparoscopic gastric sleeve procedure on January 22, 2017. She was discharged home 1-day postoperatively without any complications.

A month after surgery, she developed gradual bilateral LL numbness described as a tingling sensation, followed by LL weakness that progressed to pain. In addition, she reported short-term memory impairment. Initially, she was unable to walk without support, which gradually progressed to a complete inability to walk. There was no UL involvement. The patient reported falling down numerous times because of dizziness; however, she did not lose consciousness or have any convulsions. She also reported hoarseness of speech but no other bulbar symptoms. The patient did not have urinary or bowel changes, nor did she have visual changes. Her family history was unremarkable.

The physical examination showed that she was pale, in pain, dehydrated but conscious, oriented and alert, hemodynamically stable, and afebrile. The motor examination revealed normal LL tone, a slight decrease in power with absent reflexes, and decreased sensation up to the knees. UL reflexes were decreased. Initial laboratory test results (complete blood count, erythrocyte sedimentation rate, and C-reactive protein) were normal. Nutritional assessment showed the following results: normal vitamin B_12_ (456 *μ*mol/L), normal selenium (1.16 *μ*mol/L), low vitamin D, low vitamin B_1_ (0.28 *μ*mol/L), and normal copper (16 *μ*mol/L) levels. Her electrolyte levels were normal except for decreased potassium (K: 2.9 mmol/L). Serum protein electrophoresis demonstrated hypoalbuminemia (31 g/L).

A lumber puncture revealed clear colorless CSF with a volume of 0.5 mL and the following results: WBC 3 cells/mm^3^, RBC 107 cells/mm^3^, glucose level 4 mmol/L, and protein level 0.80 g/L. CSF viral bacterial and fungal culture results were negative. Because of the progression of her symptoms, she underwent further neurological workup. An MRI study of the dorsal spine was unremarkable, except for EMG axonal involvement. These findings were consistent with GBS.

She did not require admission to ICU but received a course of IVIG (30 g daily), nutritional replacement, vitamin B complex, and vitamin D. She started physiotherapy and was discharged from hospital in good condition.

## 5. Discussion

GBS is an inflammatory polyneuropathy that has a significant overlap in symptoms with nutritional polyneuropathies. It is an acute post-infectious autoimmune syndrome affecting the peripheral nerves and resulting in ascending symmetrical motor and sensory deficits. Patients present with ascending weakness. Cranial nerve deficits and respiratory muscle involvement occur in the later stages. Weeks prior to the onset of symptoms, patients may have a viral or bacterial infection, *Campylobacter jejuni* being the most common causative organism. Diagnosis of GBS is based on the clinical features and supporting laboratory evidence. The classic triad includes symmetric, rapidly evolving, ascending flaccid paralysis, areflexia, and elevated spinal fluid protein without cells.

Supportive care and specific therapy are the main components of managing GBS, with supportive care being the fundamental therapeutic route. The progression of neuropathy can be significantly threatening, thus giving rise to multiple complications that necessitate close monitoring, including respiratory compromise, cranial nerve dysfunction, and autonomic instability. Pain and psychological stress should not be neglected and must be managed as well as physical therapy. High-dose intravenous immunoglobulin or plasmapheresis (in severe cases) should be initiated after the diagnosis has been confirmed [7]. Approximately 85% of patients will achieve full recovery [7], but up to 15% will have a residual disability ranging from mild to severe. Causes of death include adult respiratory distress syndrome, sepsis, pulmonary emboli, and cardiac arrest (each comprising between 1% and 18% of cases) [8].  Table 1 shows case reports of bariatric surgeries that resulted in the patient developing GBS postoperatively.

All three of our cases presented with GBS-like symptoms. Their laboratory results and nutritional assessments were all normal, which ruled out nutritional deficiencies as a cause. Their EMG results and clinical findings were, however, consistent with GBS, and they improved with IVIG treatment. Such findings are consistent with a diagnosis of GBS. Chang et al. [7] presented two cases that had similar clinical presentations to the three cases in our study in that the patients all reported progressive LL weakness and showed unremarkable laboratory test results and MRI scans, but had positive findings on the EMG. They were also diagnosed with GBS from these findings and were treated with IVIG. Aluka et al. [6] also reported a case of a 40-year-old female who underwent a laparoscopic Roux-en-Y gastric bypass procedure and later an uneventful laparoscopic cholecystectomy procedure who was postoperatively diagnosed with GBS. She showed a similar clinical picture to our cases, with negative results of the neurological workup and a normal nutritional assessment. The findings of the NCSs were consistent with the axonal form of GBS. The patient was prescribed gabapentin, physical therapy, and the assistance of a walker for 6 months before her symptoms completely resolved.

On the other hand, in their cross-sectional study, Algahtani et al. [9] reported the neurological complications of bariatric surgery in 15 of 451 patients. After reviewing the patients' clinical examination, laboratory and imaging results, and therapy, the authors reported that nine patients developed peripheral neuropathy, GBS, vitamin B_12_ deficiency, copper deficiency, and Wernicke encephalopathy. Most of the patients recovered completely. The cases presented in this study show that nutritional deficiencies could be the cause of the polyneuropathies that they developed, as opposed to the other cases with normal nutritional workup results.

## 6. Conclusion

GBS is a potential delayed complication of bariatric surgery that warrants counseling preoperatively. The adverse effects of each postbariatric surgery polyneuropathy should be clearly explained to patients undergoing this type of procedure. A clear awareness program and follow-up schedules should be planned and practiced by the operating surgeon, dietician, and family physician.

## Figures and Tables

**Table 1:** Review of literature: GBS case reports after bariatric surgeries.

Name and author	Year	Country	Study type	Treatment	Outcome
Guillain-Barré-like syndrome after bariatric surgery [5] Natalia Bodunova	2015	Russia	Case report	Vitamin B complex (milgamma), thiamine, pyridoxine, and ferrous sulfate	The neurological symptoms stabilized when treatment was initiated. Over the next several months, the symptoms gradually improved; he required the assistance of a walker for 6 months before his symptoms completely resolved.
Guillain-Barré syndrome and postbariatric surgery polyneuropathies [6] Kanayochukwu J. Aluka	2009	Washington, DC, USA	Case report	Gabapentin, physical therapy	Symptoms gradually improved; she required the assistance of a walker for 6 months before her symptoms completely resolved.
Guillain-Barré syndrome (demyelinating) six weeks after bariatric surgery: a case report and literature review [10] N. Ishaque	2015	Karachi, Pakistan	Case report	Intravenous immunoglobulin (IVIG) and rehabilitation	She had complete recovery on follow-up.
Weakness after gastric bypass [7] Craig G. Chang	2002	Los Angeles and Kansas City, USA	Case series	First patient: plasmapheresis. Therapy was changed to intravenous immunoglobulin G (for 1 year) Second patient: IVIG	First patient: regained some strength in her legs but is confined to a wheelchair because of uncontrollable shaking and persistent weakness of her upper limb. Second patient: improvement in sensation and strength after second dose of IVIG was discharged to the rehabilitation unit and after 8 weeks was at her baseline premorbid ambulatory status.
Neurological complications of bariatric surgery [9] Hussein A. Algahtani	2016	Jeddah, Saudi Arabia	451 patients who underwent bariatric surgery		Fourteen patients had full recovery from the neurological signs and symptoms; one patient died.
Acute axonal polyneuropathy with predominant proximal involvement: an uncommon neurological complication of bariatric surgery [11] Flavia Costa Nunes Machado	2006	São Paulo, Brazil	Case report	Vitamin supplementation (B_1_ and B_6_ vitamin) and IVIG	Rapidly recovered proximal muscle strength.
Neurological complications of bariatric surgery [12] Anne Landais	2014	Abymes, France	Case series	First patient: IV thiamine replacement therapy, followed by intramuscular thiamine daily and physiotherapy Second patient: IV vitamin B_1_ and physiotherapy	First patient: after a few days of treatment, there was a slight improvement in gait disorders. She slowly but progressively improved after physiotherapy, and 6 months later, she had nearly completely recovered. Second patient: there was an improvement of the neurological problems following vitamin B_1_ supplementation. He was seen 5 months later; motor improvement was equally limited. The patient was able to stand and walk a few meters but needed a wheelchair for long distances.
Rare neurological complication of bariatric surgery: acute motor axonal neuropathy (AMAN), a severe motor axonal form of Guillain-Barré syndrome [13] AF Landais	2014	France	Case report	IVIG	Seven days after initiation of treatment, she was transferred to the rehabilitation department.

## References

[1] Monzo-Beltran L., Vazquez-Tarragón A., Cerdà C. (2017). One-year follow-up of clinical, metabolic and oxidative stress profile of morbid obese patients after laparoscopic sleeve gastrectomy. 8-oxo-dG as a clinical marker. *Redox Biology*.

[2] Al-Khaldi Y. (2016). Bariatric surgery in Saudi Arabia: the urgent need for standards. *Saudi Journal of Obesity*.

[3] Yin D. P., Gao Q., Ma L. L. (2011). Assessment of different bariatric surgeries in the treatment of obesity and insulin resistance in mice. *Annals of Surgery*.

[4] Angrisani L., Santonicola A., Iovino P. (2018). IFSO Worldwide Survey 2016: primary, endoluminal, and revisional procedures. *Obesity Surgery*.

[5] Bodunova N., Degterev D., Suponeva N., Zorin E. (2015). Guillain-Barré-like syndrome after bariatric surgery. *Bariatric Times*.

[6] Aluka K. J., Turner P. L., Fullum T. M. (2009). Guillain-Barré syndrome and postbariatric surgery polyneuropathies. *JSLS: Journal of the Society of Laparoendoscopic Surgeons*.

[7] Chang C. G., Helling T. S., Black W. E., Rymer M. M. (2002). Weakness after gastric bypass. *Obesity Surgery*.

[8] Bhagat H., Dash H., Chauhan R., Khanna P., Bithal P. (2014). Intensive care management of Guillain-Barre syndrome: a retrospective outcome study and review of literature. *Journal of Neuroanaesthesiology & Critical Care*.

[9] Algahtani H. A., Khan A. S., Khan M. A., Aldarmahi A. A., Lodhi Y. (2016). Neurological complications of bariatric surgery. *Neurosciences*.

[10] Ishaque N., Khealani B. A., Shariff A. H., Wasay M. (2015). Guillain–Barré syndrome (demyelinating) six weeks after bariatric surgery: a case report and literature review. *Obesity Research & Clinical Practice*.

[11] Machado F. C. N., Valério B. C. O., Morgulis R. N. F., Nunes K. F., Mazzali-Verst S. (2006). Acute axonal polyneuropathy with predominant proximal involvement: an uncommon neurological complication of bariatric surgery. *Arquivos de Neuro-Psiquiatria*.

[12] Landais A. (2014). Neurological complications of bariatric surgery. *Obesity Surgery*.

[13] Landais A. F. (2014). Rare neurologic complication of bariatric surgery: acute motor axonal neuropathy (AMAN), a severe motor axonal form of the Guillain Barré syndrome. *Surgery for Obesity and Related Diseases*.

